# Conjugation of Triterpenic Acids with 3-Aminoquinuclidine Moiety: An Approach to Acetylcholinesterase Mixed or Uncompetitive Type Inhibitors

**DOI:** 10.3390/molecules30010095

**Published:** 2024-12-29

**Authors:** Anastasiya V. Petrova, Ha T. T. Nguyen, Irina V. Zueva, Konstantin A. Petrov, Alexander N. Lobov, Oxana B. Kazakova

**Affiliations:** 1 Ufa Institute of Chemistry, Ufa Federal Research Centre, Russian Academy of Science, 71, Prospect Octyabrya, Ufa 450054, Russia; lobovan@anrb.ru (A.N.L.); obf@anrb.ru (O.B.K.); 2 Institute of Chemistry, Vietnam Academy of Science and Technology (VAST), 18, Hoang Quoc Viet Road, Cau Giay, Ha Noi 10000, Vietnam; thuha.vast@gmail.com; 3 Arbuzov Institute of Organic and Physical Chemistry, FRC Kazan Scientific Center, Russian Academy of Science, 8, Arbuzov Street, Kazan 420088, Russia; zueva.irina.vladimirovna@gmail.com (I.V.Z.); kpetrov2005@mail.ru (K.A.P.)

**Keywords:** triterpenoids, lupane, oleanane, ursane, 3-aminoquinuclidine, acetylcholinesterase, Alzheimer’s disease

## Abstract

Alzheimer’s disease (AD) poses a significant public health issue. Despite the fact that today there are several methods of maintenance therapy, one of the most widely used methods is designed to correct the deficiency of acetylcholine. In the search for new potential inhibitors of cholinesterase enzymes, eight new derivatives of 3-oxo- or 2,3-indolo-triterpenic acid conjugated with amino-quinuclidine bicyclic cores were designed and synthesized. Then, the obtained compounds were screened in Ellman’s assays for their ability to inhibit acetylcholinesterase enzyme, and for each of the active compounds, the type of inhibition was determined. The obtained results demonstrate the dependence of the activity on the triterpenoid structure and the type of substituents. The best activity for ursolic acid derivatives was observed for the 3-oxoamide **8**, with an IC_50_ value of 0.43 µM, acting as a mixed-type inhibitor. In turn, for the oleanane type, the amide with an indole unit in the A ring **11** exhibited the best activity with an IC_50_ value of 0.47 µM (while the ursane-type analog was weakly active) and led to an uncompetitive type of inhibition. Thus, 3-amidoquinuclidine-triterpenoids conjugates could be considered novel inhibitors of acetylcholinesterase with a different mechanism of action.

## 1. Introduction

Nature has been a source of inspiration and resources for humanity for thousands of years. Among all the diversity in nature, a special place is occupied by natural compounds, biologically active substances found in plants, animals, and bacteria. These compounds, often called phytonutrients, have unique properties that can have a significant impact on human health. Modern scientific research increasingly confirms the importance of natural compounds in the prevention and treatment of various diseases, including cancer, malaria, neurodegenerative diseases, and many others [1,2,3].

Pentacyclic triterpenoids are one of the most extensively researched classes of secondary metabolites with interest in terms of their structure and spectrum of biological activity. Their potential applications as new therapeutic agents are being subjected to clinical trials. Thus, 20% betulinic acid ointment was evaluated for the safety and effectiveness of an experimental treatment for dysplastic nevi with the potential to transform into melanoma (ClinicalTrials.gov; NCT00346502, 28 June 2021) [4]. Oleogel-S10, whose active ingredient is betulin, is superior to Octenilin^®^ wound gel in the treatment of superficial, partially healed burn wounds [5]. Glycyrrhizic acid (GZA) was studied in clinical trials to assess its potential anti-depressive effect in combination with SSRIs (selective serotonin reuptake inhibitors) on 56 patients with MDD (Major depressive disorder) [6]. Current results appear to demonstrate that adjunctive glycyrrhizic acid therapy leads to impressively greater symptomatic improvement, better treatment response, and a faster onset of clinical effects regarding depressive patients. 18β-Glycyrrhetinic acid (GA) is obtained from GZA by removing two glucuronic acid groups [7], and dexamethasone was tested in phase IV clinical trials to treat immunological thrombocytopenia (ITP) (ClinicalTrials.gov; NCT03998982, 11 June 2021) [8].

Today, triterpenoids undergo modification of the basic skeleton in multiple ways, allowing the formation of various derivatives that also have pharmacological potential, such as anticancer [9], antiviral [10], antimicrobial [11], and, in particular, enzyme inhibitory properties [12]. Both native and semi-synthetic derivatives show great potential in this area. For example, it is known that oleanolic acid (OA) shares mechanisms of action with metformin for the treatment of diabetes [13]. Therefore, OA is presently under evaluation in phase II studies in the framework of the OLTRAD (OLeanolic acid TReAtment for type 2 Diabetes) study, which has been designed to demonstrate that the regular intake of an OA-enriched functional olive oil is effective as an adjuvant to metformin antidiabetic drug therapy. The hypothesis is that the inclusion of this functional olive oil in the diet will enhance the effects of the pharmacological treatment in diabetic patients, and may even reduce the need for prescriptions of such medications (ClinicalTrials.gov; NCT06030544, 11 September 2023).

**Figure 1:**
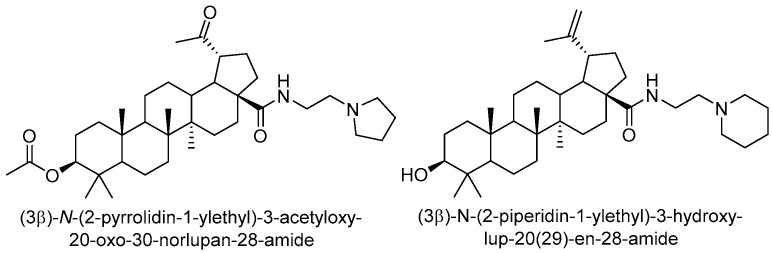
Pyrrolidine and piperidinyl-containing derivatives of betulinic acid [16].

It is also known that natural compounds and their derivatives form the basis of many drugs aimed at inhibiting enzymes of the cholinesterase family [14]. In view of this, a large number of natural compounds have been modified to enhance or redirect mechanisms of cholinesterase inhibiting and changing functional properties. For example, OA modified at the C3 position in one step to diethoxyphosphoryloxy ester was characterized as a nanomolar inhibitor of butyrylcholinesterase (K 6.59 nM) [15]. Based on betulinic acid, a derivative containing the cyclic amine pyrrolidine in position C28 was obtained, possessing inhibitory properties against BChE with K_i_ values of 0.07 µM (Figure 1). At the same time, it was interesting that the replacement of the pyrrolidine unit with piperidinyl led to selective inhibition of AChE (K_i_ 1.00 µM) [16] (Figure 1). Thus, one-step methods of conjugation with various pharmacophores are simple, universal, and relatively cheap ways of regulating biological properties, and therefore it is important to continue these studies. In accordance with this, the direction of conjugation of native molecules with other natural units began to develop. For example, betulinic acid-diosgenin conjugate showed a selective cytotoxicity against human T-lymphoblastic leukemia cancer cells (IC_50_ 6.5 µM), in contrast to parent diosgenin, which does not exhibit cytotoxicity [17]. The conjugates of triterpenic acid with 1,3- or 1,4 diazabicyclo [3.2.2] nonanes, which are structurally close to alkaloids, proved to be as excellent inhibitors of the enzyme butyrylcholinesterase (BChE) acting as hyperbolic mixed-type inhibitor [18].

**Figure 2:**
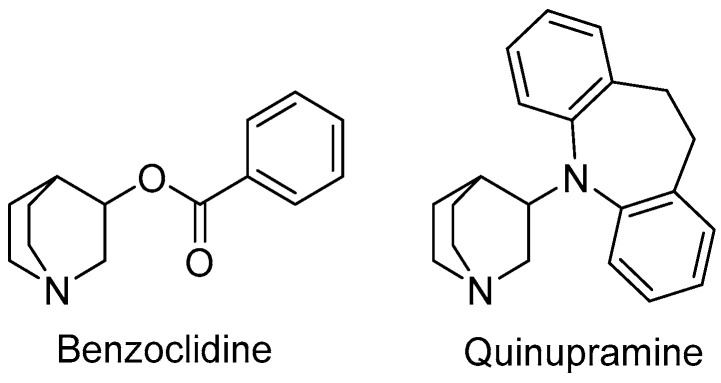
The structure of Benzoclidine and Quinupramine.

Quinuclidine is a molecule with a bicyclic structure containing a nitrogen bridge, which makes it versatile in the design of new drugs and materials in the field of medicinal chemistry. Quinuclidine is found in nature as a component of alkaloids, for example, it is a structural unit of quinine, ajmaline, and cinchonamine. The quinuclidine moiety is used to prepare various therapeutically potent derivatives, such as the approved drugs Benzoclidine and Quinupramine, which are drugs for the treatment of neurotic disorders and depression (Figure 2). Quinuclidine derivatives have been studied for their various pharmacological properties, including antimalarial, anticonvulsant, and antihistaminic activities. For example, the transformation of amino/amido quinuclidine to quaternary ammonium salts leads to compounds with antimicrobial activity [19,20]. It was also shown that *N*-benzyl-3-benzamidoquinuclidine exhibited the butyrylcholinesterase inhibitory activity with K_i_ = 3.7 µM [21].

Among a wide range of different neurodegenerative diseases, Alzheimer’s disease (AD) has a special place in its negative significance for society. Currently, about five drugs are used to relieve the symptoms of AD, most of which have a pronounced cholinomimetic effect and are aimed at improving cognitive abilities reduced due to hypofunction of the cholinergic neurotransmitter system [22]. Cholinesterase inhibitors are historically the first and most developed group of drugs proposed for the treatment of AD. The clinical effect of these drugs currently used is moderate and appears rather slowly, while the side effects are more pronounced and limit their use.

Taking into account the above, here we present the first investigation of triterpenic acid-quinuclidine conjugates as an acetylcholinesterase inhibitor.

## 2. Results and Discussion

**Figure 3:**
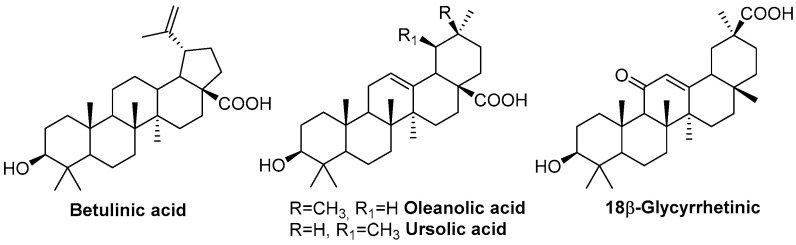
Native pentacyclic triterpenic acids.

In our study, natural pentacyclic triterpenic betulinic, oleanolic, ursolic, and 18β-glycyrrhetinic acids were used as starting platforms (Figure 3). These four acids are naturally found in a large variety of vegetables foods, medicinal herbs, and plants. Betulinic acid was obtained by the oxidation of betulin, isolated from birch bark. Oleanolic and ursolic acids can be extracted from a number of edible and medicinal botanicals, such as *Mirabilis jalapa*, *Olea europaea*, rosemary, apples, basil, etc. 18β-Glycyrrhetinic acid was isolated by hydrolysis of glycyrrhizic acid, which is the main active component of *Glycyrrhíza glábra*. All these compounds play a significant role in medicinal chemistry and biotechnology, with ongoing research exploring their therapeutic and industrial potential. In this regard, these compounds were selected for further modifications.

**Scheme 1:**
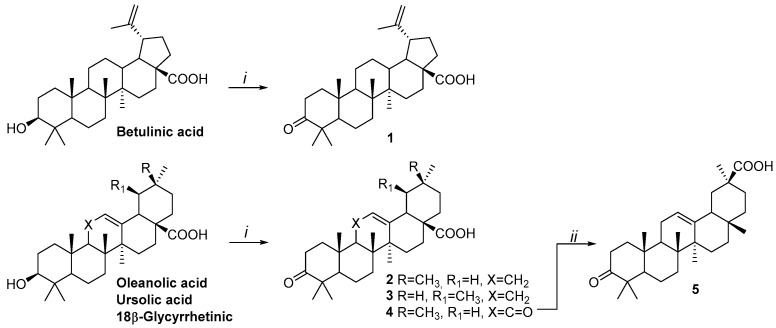
Synthesis of 3-oxo analogs. Reagent and conditions: *i*. Jones reagent, acetone, 25 °C, 1 h; *ii*. Zn/HCl, 1,4-dioxane, 25 °C, 24 h.

3-Oxo-acids of lupane **1**, oleanane **2**, **4**, and ursane **3** types were obtained by Jones oxidation of their parent compound [23]. 11-Deoxo-glycyrrhetinic acid **4** was synthesized by reduction of the 11-oxo-group using Zn/HCl (Scheme 1).

**Scheme 2:**
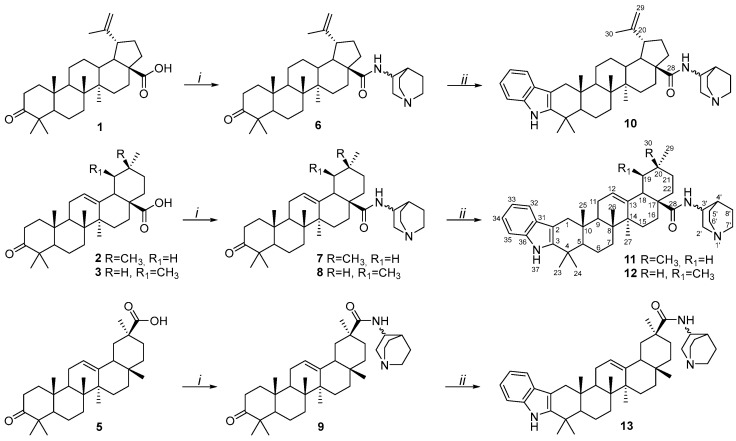
Synthesis of lupane-, oleanane-, and ursane-type 3-aminoquinuclidine derivatives. Reagent and conditions: *i*. (1) (COCl)_2_, Et_3_N, CH_2_Cl_2_, 25 °C, 2 h; (2) 3-aminoquinuclidine dihydrochloride, Et_3_N, CH_2_Cl_2_, reflux, 4 h; *ii*. PhNHNH_2_, AcOH, reflux, 4 h.

Next, compounds **1**–**3** and **5** reacted with (±)-3-aminoquinuclidine dihydrochloride by the acyl chloride method in the presence of Et_3_N to afford amides **6**–**8** with yields of 75–80% (Scheme 2).

The indole scaffold is one of the actively developing structural units in medicinal and organic chemistry. Indole-containing molecules are often found in natural products and exhibit a wide range of biological activities, such as antibacterial, antitumor, antiplasma, and cholinesterase inhibitory effects [24]. For example, physostigmine and its analogs are used as drugs to treat myasthenia and glaucoma [25]. Based on these data, we decided to synthesize indole analogs of the obtained amides. For this purpose, amides **6**–**9** were involved in Fischer indolization with phenylhydrazine in acetic acid, which resulted in the production of derivatives **10**–**13** (yields 90–95%).

**Figure 4:**
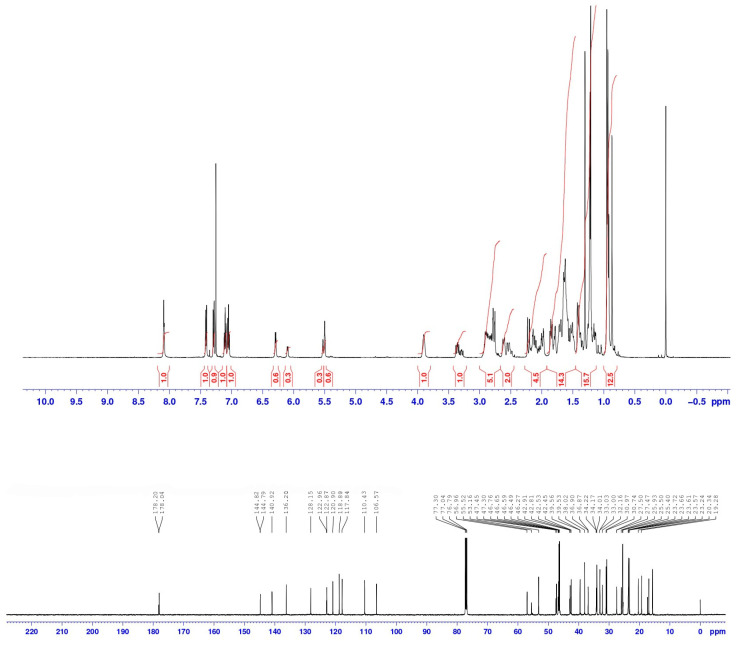
An example of ^1^H and ^13^C spectra of compound **11**.

The structure of the obtained derivatives was confirmed by ^1^H and ^13^C NMR spectroscopy. Thus, the ^13^C NMR spectra of new compounds contain the signal at δ ~178 ppm, characteristic of the amide function carbon atom at C28. Indole derivatives **10**–**13** were also characterized by the presence of signals of aromatic protons at δ 7–8 ppm in ^1^H NMR spectra (Figure 4). For full spectral data, see Supplementary Information.

All synthesized derivatives **6**–**13** were subjected to Ellman’s assays to determine their ability to inhibit the AChE enzyme (Table 1). Donepezil was used as a positive control since it is the most effective and selective AChE inhibitor. It is also the most commonly prescribed cholinesterase inhibitor for the treatment of Alzheimer’s disease and has been approved by the FDA since 1996. Among 3-oxo-derivatives, compound **8** was found to be the most active compound with IC_50_ 0.43 µM, being only 14.8 times less active than donepezil. Compound **7** was also identified as a good inhibitor with IC_50_ 1.67 µM. Amides of betulinic **6** and glycyrrhetinic **9** acids showed weak activity (IC_50_ 17.0 µM and 29 µM, respectively). The introduction of an indole ring into the molecule had a positive effect only on derivatives **6** and **7**. Thus, for the amide of indolobetulinic acid **10,** the IC_50_ value was 4.77 µM, which is 3.5 times more active than its 3-oxo-analog **6**. The same increase in activity was observed for compound **11** which exhibited strong activity with IC_50_ 0.47 µM, being 3.5 times more active than **7**. Compounds **12** and **13** showed weak activity, which indicates a negative influence of the indole cycle.

In vitro inhibition of recombinant human AChE by the most active compounds **8** and **11** was investigated. As shown in Table 2, the IC_50_ value for lead compounds were 5.33 µM for **8**, and 8.89 µM for **11**.

**Figure 5:**
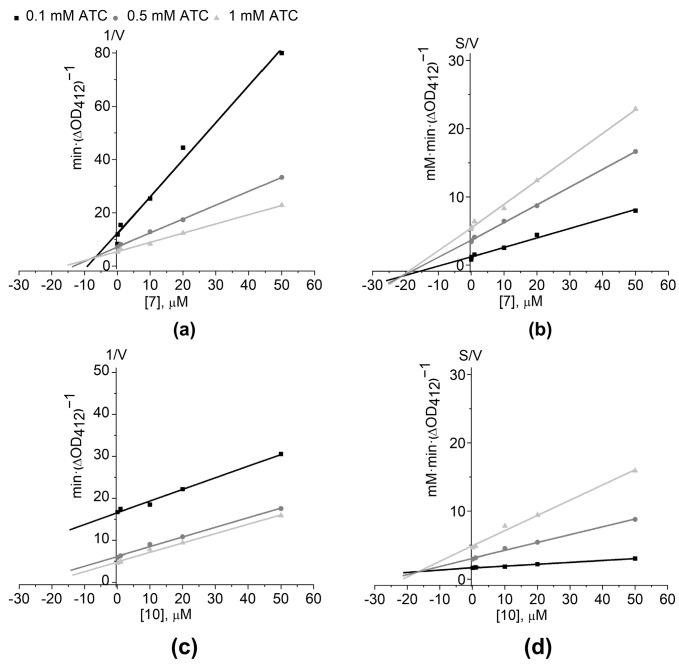
Determination of reversible inhibition type and inhibition constants of hAChE by compound **8** (**a**,**b**) and **11** (**c**,**d**): (**a**,**c**) Dixon plot; (**b**,**d**) Cornish-Bowden plot for the inhibition.

To study the mechanism of hAChE inhibition by compounds **8** and **11**, the inhibition constants were determined (Table 3). Experiments were performed using three different concentrations of ATC as substrate. For **8**, analyses of Dixon and Cornish-Bowden plots established that AChE inhibition was of mixed type, with K_ci_ (competitive inhibition) and K_ui_ (uncompetitive) components: K_ci_ = 6.8 ± 0.4 μM, K_ui_ = 22.8 ± 1.5 μM for AChE (Figure 5).

It is known that native triterpenic acids are characterized by a mixed (for UA) and competitive (for OA) type of inhibition of AChE, while inactivity is reported for BChE [26]. The introduction of diazabicyclo [3.2.2] nonane substitute through an amide bond led to a change in the type of inhibited enzyme to BChE enzyme, and weak inhibition of AChE [18]. In our case, the introduction of a close carbocyclic amine into 3-oxo-ursolic acid resulted in inhibition of AChE, also acting as a mixed-type inhibitor. On the other hand, for oleanane-type derivatives with an indole cycle in the A ring, a change in the mechanism of action was observed. The analysis of Dixon and Cornish-Bowden plots of compound **11** established that AChE inhibition was of uncompetitive type with K_ui_ = 19.1 ± 5.1 μM (Figure 5). These data suggest the influence of the indole ring on activity and the mechanism of action, in accordance with the fact that a weak activity was observed for the ursolic acid indoloamide **11**. Thus, we observe the differences in the activity of the isomeric oleanolic and ursolic acids holding 3-oxo- or 2,3-indole substituents.

## 3. Materials and Methods

### 3.1. Chemistry

The spectra were recorded at the Center for the Collective Use ‘Chemistry’ of the Ufa Institute of Chemistry of the UFRC RAS and RCCU “Agidel” of the UFRC RAS. ^1^H and ^13^C NMR spectra were recorded on a “Bruker Avance-III” (Bruker, Billerica, MA, USA, 500 and 125.5 MHz, respectively, δ, ppm, Hz) in CDCl_3_, internal standard—tetramethylsilane. Melting points were detected on a microtable «Rapido PHMK05» (Nagema, Dresden, Germany). Thin-layer chromatography analyses were performed on Sorbfil plates (Sorbpolimer, Krasnodar, Russia), using the solvent system chloroform–ethyl acetate, 40:1. Substances were detected by spraying the plates with a 10% sulfuric acid solution followed by subsequent heating at 100–120 °C for 2–3 min. All reagents purchased from commercial sources were chemically or analytically pure. These reagents were used directly, except for anhydrous solvents having been dried according to standard procedures prior to performing synthetic reactions. The analytical data obtained for the known compounds **1**–**4** were in agreement with those reported in the literature [23].

General synthesis of compounds **5**–**8**. To a solution of compound 1 (1 mmol; 0.45 g), 2 (1 mmol; 0.45 g), 3 (1 mmol; 0.45 g), or **4** (1 mmol; 0.45 g), in dry CH_2_Cl_2_ (15 mL), (COCl)_2_ (0.08 mL, 1 mmol) and Et_3_N (2 drops) were added. The reaction mixture was stirred at room temperature for 2 h, then CH_2_Cl_2_ was evaporated to give an intermediate acyl chloride. This residue was dissolved in CH_2_Cl_2_ (5 mL) and a CH_2_Cl_2_ solution of (±)-3-aminoquinuclidine dihydrochloride (1.3 mmol; 0.26 g) and Et_3_N (1 mmol; 0.14 mL) was added in each reaction. The reaction mixture was refluxed for 2 h, the organic layer was diluted with cold water (20 mL) and separated. The aqueous layer was extracted with CH_2_Cl_2_ (2 × 15 mL), the combined extracts were washed with 5% HCl (3 × 15 mL), H_2_O (2 × 15 mL), dried over CaCl_2_, and the solvent was evaporated in a water jet vacuum. Purification of the crude product was performed using column chromatography on SiO_2_ by elution with a mixture of CHCl_3_-EtOH (5:1).

*3-oxo-lup-20-en-28-((±)-3-aminoquinuclidine) amide* (**5**). Yield 0.51 g (90%), beige powder, mp: 137.8 °C. ^1^H NMR (CDCl_3_, δ ppm, *J* Hz): 0.86, 0.88, 0.91, 0.96, 1.00, 1.62 (18H, s, 6CH_3_), 1.10–3.05 (36H, m, CH and CH_2_), 3.50–4.00 (1H, m, CH), 4.55 and 4.65 (2H, both s, H-29), 8.20 (1H, NH). ^13^C NMR (CDCl_3_, δ, ppm): 22.17, 24.43, 24.97, 25.50, 25.56, 26.43, 26.58, 26.61, 29.40, 30.76, 32.89, 33.05, 33.99, 34.03, 36.80, 37.23, 37.98, 38.47, 39.49, 40.58, 42.31, 44.89, 45.15, 46.48, 47.18, 49.91, 49.96, 50.14, 52.74, 53.86, 54.84, 55.65, 57.12, 109.23 (C29), 150.81 (C20), 177.50 (C28), 218.12 (C3). MW 562.88. C_37_H_58_N_2_O_2_. Calculated, %: C 78.95; H 10.39; N 4.98.
*3-oxo-olean-12-en-28-((±)-3-aminoquinuclidine) amide* (**6**). Yield 0.50 g (89%), beige powder, mp: 154.7 °C. ^1^H NMR (CDCl_3_, δ ppm, *J* Hz): 0.81, 0.84, 0.91, 0.92, 1.03, 1.08, 1.17 (s, 21H, 7CH_3_), 1.30–3.05 (m, 34H, CH and CH_2_), 3.20–4.00 (m, 1H, CH), 5.35 (s, 1H, H-12), 8.00 (1H, NH). ^13^C NMR (CDCl_3_, δ, ppm): 23.61, 23.82, 25.39, 25.58, 25.66, 26.46, 26.58, 27.41, 30.66, 30.72, 32.17, 32.98, 33.06, 33.17, 34.14, 34.36, 36.72, 36.76, 39.30, 39.41, 42.30, 42.38, 42.58, 45.50, 46.10, 46.37, 46.60, 46.48, 46.80, 47.16, 47.42, 53.18, 55.27, 122.25 (C12), 144.99 (C13), 178.09 (C28), 217.50 (C3). MW 562.88. C_37_H_58_N_2_O_2_. Calculated, %: C 78.95; H 10.39; N 4.98.
*3-oxo-urs-12-en-28-((±)-3-aminoquinuclidine) amide* (**7**). Yield 0.49 g (88%), white powder, mp: 170.6 °C. ^1^H NMR (CDCl_3_, δ ppm, *J* Hz): 0.85, 0.86, 0.90, 0.91, 0.99, 1.05, 1.06 (21H, s, 7CH_3_), 1.18–2.50 (34H, m, CH and CH_2_), 3.50–3.90 (m, 1H, CH), 5.38 (s, 1H, H-12), 7.80 (1H, NH). ^13^C NMR (CDCl_3_, δ, ppm): 18.39,18.74, 19.53, 21.18, 21.40, 22.18, 22.26, 23.09, 23.28, 23.44, 23.97, 24.53, 24.95, 27.95, 30.95, 32.49, 34.09, 36.59, 37.43, 38.56, 39.18, 39.43, 39.48, 42.29, 45.17, 46.65, 47.28, 47.73, 52.36, 52.68, 53.83, 55.07, 57.59, 124.92 (C12), 139.24 (C13), 178.82 (C28), 217.75 (C3). MW 562.88. C_37_H_58_N_2_O_2_. Calculated, %: C 78.95; H 10.39; N 4.98.
*3-oxo-11-dezoxo-olean-12-en-30-((±)-3-aminoquinuclidine) amide* (**8**). Yield 0.51 g (91%), white powder, mp: 152.8 °C. ^1^H NMR (CDCl_3_, δ ppm, *J* Hz): 0.90, 0.98, 1.00, 1.05, 1.15. 1.18, 1.18 (s, 21H, 7CH_3_), 1.20–2.60 (m, 34H, CH and CH_2_), 3.00–4.40 (m, 1H, CH), 5.30 (s, 1H, H-12), 7.60 (1H, NH). ^13^C NMR (CDCl_3_, δ, ppm): 25.81, 26.11, 26.49 (2C), 26.97 (2C), 28.30, 28.53, 29.39, 29.47, 31.45, 31.99, 32.18, 34.20, 36.68, 38.31, 38.52, 39.31, 39.74, 41.72, 43.12, 43.25, 43.88, 44.49, 46.24, 46.31, 46.84, 47.44, 47.73, 47.93, 50.50 (2C), 55.30, 122.37 (C12), 144.43 (C13), 178.01 (C30), 217.78 (C3). MW 562.88. C_37_H_58_N_2_O_2_. Calculated, %: C 78.95; H 10.39; N 4.98.

General synthesis of compounds 9–12. A mixture of **5** (1 mmol; 0.56 g), **6** (1 mmol; 0.56 g), **7** (1 mmol; 0.56 g), or **8** (1 mmol; 0.56 g) and phenylhydrazine (0.42 mL, 3.5 mmol) in glacial acetic acid (20 mL) was heated at reflux for 15 h. After cooling, the reaction mixture was poured into water, the residue was filtered off under suction, washed with water and dried, and then purified by column chromatography on SiO_2_ by elution with a mixture of CHCl_3_-EtOH (10:1).

*[3,2b]Indolo-lup-20-en-28-((±)*-*3-aminoquinuclidine) amide* (**9**). Yield 0.58 g (91%), brown powder, mp: 115.6 °C. ^1^H NMR (MeOD, δ ppm, *J* Hz): 0.86 (s, 3H, H-25), 1.00 (s, 3H, H-26), 1.04 (s, 3H, H-27), 1.12 (m, 1H, H_ax_-12), 1.20 (s, 3H, H-24), 1.25 (m, 1H, H_eq_-15), 1.31 (s, 3H, H-23), 1.39 (dd, 1H, ^3^*J*_5–6ax_ = 10.8, ^3^*J*_5–6eq_ = 3.5, H-5), 1.39 (m, 1H, H_ax_-15), 1.39 (m, 1H, H_ax_-11), 1.40 (m, 1H, H_eq_-21), 1.47 (m, 1H, H_eq_-7), 1.49 (m, 1H, H_ax_-22), 1.51 (m, 1H, H_ax_-7), 1.56 (m, 1H, H_ax_-6), 1.60 (m, 1H, H_ax_-16), 1.61 (dd, 1H, ^3^*J*_9–11ax_ = 13.0, ^3^*J*_9–11eq_ = 3.0, H-9), 1.62 (m, 1H, H_eq_-6), 1.63 (t, 1H, ^3^*J*_18–19_ = 11.2, ^3^*J*_18–13_ = 11.2, H-18), 1.66 (m, 1H, H_anti_-5′), 1.71 (s, 3H, H-30), 1.72 (m, 1H, H_eq_-11), 1.81 (m, 1H, H_eq_-12), 1.88 (m, 2H, H-8′), 1.89 (m, 1H, H_ax_-21), 1.90 (m, 1H, H_eq_-22), 1.98(m, 1H, H_syn_-5′), 2.15 (d, 1H, ^2^*J* = 14.9, H_A_-1), 2.18 (m, 1H, H-4′), 2.24 (m, 1H, H_eq_-16), 2.60 (dd, 1H, ^3^*J*_13–12ax_ = 13.3, ^3^*J*_13–18_ = 11.2, ^3^*J*_13–12eq_ = 3.6, H-13), 2.83 (d, 1H, ^2^*J* = 14.9, H_B_-1), 3.00 (m, 2H, H-6′), 3.01 (m, 2H, H-7′), 3.11 (td, 1H, ^3^*J*_19–18_ = 11.2, ^3^*J*_19–21ax_ = 11.2, ^3^*J*_19–21eq_ = 4.9, H-19), 3.17 (m, 1H, H_trans_-2′), 3.41 (m, 1H, H_cis_-2′), 4.22 (m, 1H, H-3′), 4.62 (d, 1H, ^2^*J* = 2.1, H_A_-29), 4.76 (d, 1H, ^2^*J* = 2.1, H_B_-29), 7.01 (ddd, 1H, ^3^*J*_33–32_ = 7.6, ^3^*J*_33–34_ = 7.1, ^3^*J*_33–35_ = 1.1, H-33), 7.07 (ddd, 1H, ^3^*J*_34–35_ = 8.0, ^3^*J*_34–33_ = 7.1, ^3^*J*_34–32_ = 1.1, H-34), 7.31 (dd, 1H, ^3^*J*_35–34_ = 8.0, ^3^*J*_35–33_ = 1.1, H-35), 7.37 (dd, 1H, ^3^*J*_32–33_ = 7.6, ^3^*J*_32–34_ = 1.1, H-32). ^13^C NMR (MeOD, δ ppm): 14.84 (C27); 16.10 (C26); 16.51 (C25); 18.00 (C5′); 19.50 (C6); 19.52 (C30); 21.81 (C11); 22.34 (C8′); 22.96 (C24); 25.14 (C4′); 26.02 (C12); 29.74 (C15); 30.71 (C23); 31.01 (C21); 33.20 (C16); 33.91 (C7), 34.43 (C4), 37.60 (C1), 38.01 (C13), 38.21 (C22), 38.56 (C10), 41.08 (C8), 42.63 (C14), 44.50 (C3′), 46.34 (C7′), 46.57 (C6′), 46.94 (C19), 49.76 (C9), 50.53 (C18), 51.43 (C2′), 53.70 (C5), 55.98 (C17), 106.42 (C2), 109.60 (C29), 110.70 (C35), 117.83 (C32), 118.55 (C33), 120.67 (C34), 128.39 (C31), 136.63 (C36), 141.60 (C3), 151.13 (C20), 178.03 (C28). MW 635.98. C_43_H_61_N_3_O. Calculated, %: C 81.21; H 9.67; N 6.61.
*[3,2b]Indolo-olean-12-en-28-((±)-3-aminoquinuclidine) amide* (**10**). Yield 0.56 g (89%), brown powder, mp: 181.6 °C. ^1^H NMR (CDCl_3_, δ ppm, *J* Hz): 0.87 (s, 3H, H-26), 0.93 (s, 3H, H-29), 0.95 (s, 3H, H-30), 0.95 (s, 3H, H-25), 1.15 (m, 1H, H_eq_-15), 1.21 (s, 3H, H-24), 1.22 (m, 1H, H_eq_-21), 1.22 (s, 3H, H-27), 1.25 (m, 1H, H_eq_-19), 1.30 (s, 3H, H-23), 1.38 (m, 1H, H_ax_-21), 1.41 (m, 1H, H_eq_-7), 1.41 (dd, 1H, ^3^*J*_5–6ax_ = 10.8, ^3^*J*_5–6eq_ = 2.5, H-5), 1.50 (m, 1H, H*_anti_*-5′), 1.53 (m, 1H, H_ax_-6), 1.60 (m, 1H, H_ax_-7), 1.62 (m, 1H, H_ax_-22), 1.62 (m, 2H, H-8′), 1.63 (m, 1H, H_eq_-6), 1.63 (m, 1H, H*_syn_*-5′), 1.64 (m, 1H, H_ax_-15), 1.69 (m, 1H, H_eq_-22), 1.70 (m, 1H, H_eq_-16), 1.78 (sext., 1H, ^3^*J*_4′-3′_ = 3.1, ^3^*J*_4′-5′*anti*_ = 3.1, ^3^*J*_4′-5′syn_ = 3.1, ^3^*J*_4′-8′*anti*_ = 3.1, ^3^*J*_4′-8′syn_ = 3.1, H-4′), 1.83 (dd, 1H, ^2^*J* = 13.1, ^3^*J*_19ax-18_ = 14.1, H_ax_-19), 1.85 (dd, 1H, ^3^*J*_9–11ax_ = 10.9, ^3^*J*_9–11eq_ = 6.9, H-9), 2.00 (m, 1H, H_ax_-16), 2.09 (ddd, 1H, ^2^*J* = 18.5, ^3^*J*_11ax-9_ = 10.9, ^3^*J*_11ax-12_ = 3.5, H_ax_-11), 2.16 (ddd, 1H, ^2^*J* = 18.5, ^3^*J*_11eq-9_ = 6.9, ^3^*J*_11eq-12_ = 3.5, H_eq_-11), 2.21 (d, 1H, ^2^*J* = 15.1, H_A_-1), 2.54 (ddd, 1H, ^2^*J* = 14.2, ^3^*J*_2′*trans*-3′_ = 4.4, ^3^*J*_2′*trans*-7′*anti*_ = 2.4, H*_trans_*-2′), 2.62 (dd, 1H, ^3^*J*_18–19ax_ = 14.1, ^3^*J*_18–19eq_ = 4.1, H-18), 2.77 (d, 1H, ^2^*J* = 15.1, H_B_-1), 2.82 (m, 2H, H-7′); 2.90 (m, 2H, H-6′), 3.36 (ddd, 1H, ^2^*J* = 14.2, ^3^*J*_2′*cis*-3′_ = 9.1, ^3^*J*_2′*cis*-6′*anti*_ = 2.2, H*_cis_*-2′), 3.90 (ddddd, 1H, ^3^*J*_3′-2′*cis*_ = 9.1, ^3^*J*_3′-NH_ = 6.1, ^3^*J*_3′-2′*trans*_ = 4.4, ^3^*J*_3′-4′_ = 3.1 ^4^*J*_3′-5′*anti*_ = 1.5, H-3′), 5.50 (t, 1H, ^3^*J*_12–11ax_ = 3.5, ^3^*J*_12–11eq_ = 3.5, H-12), 6.29 (br.d, 1H, ^3^*J*_NH-3′_ = 6.1, NH), 7.05 (ddd, 1H, ^3^*J*_33–32_ = 7.7, ^3^*J*_33–34_ = 7.0, ^4^*J*_33–35_ = 1.0, H-33), 7.10 (ddd, 1H, ^3^*J*_34–35_ = 8.0, ^3^*J*_34–33_ = 7.0, ^4^*J*_34–32_ = 1.1, H-34), 7.29 (dd, 1H, ^3^*J*_35–34_ = 8.0, ^4^*J*_35–33_ = 1.0, H-35), 7.41 (dd, 1H, ^3^*J*_32–33_ = 7.7, ^4^*J*_32–34_ = 1.1, H-32), 8.09 (br.s, 1H, H-37). ^13^C NMR (CDCl_3_, δ ppm): 15.69 (C25), 16.90 (C26), 19.28 (C6), 20.34 (C5′), 23.24 (C24), 23.61 (C30), 23.66 (C11), 23.72 (C16), 25.40 (C27), 25.50 (C4′), 25.93 (C8′), 27.47 (C15), 30.74 (C20), 30.97 (C23), 32.16 (C7), 33.00 (C29), 33.03 (C22), 34.01 (C4), 34.17 (C21), 36.86 (C1), 38.02 (C10), 39.53 (C8), 42.45 (C14), 42.81 (C18), 46.27 (C3′), 46.27 (C9), 46.49 (C17), 46.50 (C7′), 46.59 (C19), 47.30 (C6′), 53.16 (C5), 56.96 (C2′), 106.57 (C2), 110.43 (C35), 117.84 (C32), 118.80 (C33), 120.90 (C34), 122.87 (C12), 128.15 (C31), 136.20 (C36), 140.92 (C3), 144.79 (C13), 178.04 (C28). *F1*-projection in {^1^H, ^15^N} HMBC (CDCl_3_, δ ppm): 21.12 (N1′), 117.24 (N37), 123.49 (NH). MW 635.98. C_43_H_61_N_3_O. Calculated, %: C 81.21; H 9.67; N 6.61.
*[3,2b]Indolo-urs-12-en-28-((±)-3-aminoquinuclidine) amide* (**11**). Yield 0.56 g (89%), brown powder, mp: 115.4 °C. ^1^H NMR (CDCl_3_, δ ppm, *J* Hz): 0.88 (s, 3H, H-26), 0.94 (d, 3H, ^3^*J*_29–19_ = 6.4, H-29), 0.96 (s, 3H, H-25), 0.97 (d, 3H, ^3^*J*_30–20_ = 6.4, H-30), 1.00 (m, 1H, H-20), 1.15 (m, 1H, H_eq_-15), 1.16 (s, 3H, H-27), 1.22 (s, 3H, H-24), 1.31 (s, 3H, H-23), 1.33 (m, 1H, H_ax_-21), 1.41 (dd, 1H, ^3^*J*_5–6ax_ = 11.4, ^3^*J*_5–6eq_ = 2.4, H-5), 1.44 (m, 1H, H_eq_-7), 1.45 (m, 1H, H_ax_-22), 1.48 (m, 1H, H*_anti_*-5′), 1.51 (m, 1H, H-19), 1.51 (m, 1H, H_ax_-6), 1.52 (m, 1H, H_eq_-21), 1.62 (m, 1H, H_ax_-7), 1.63 (m, 1H, H_eq_-6), 1.66 (m, 2H, H-8′), 1.67 (m, 1H, H*_syn_*-5′), 1.75 (m, 1H, H_ax_-15), 1.80 (m, 1H, H_eq_-16), 1.83 (dd, 1H, ^3^*J*_9–11ax_ = 10.6, ^3^*J*_9–11eq_ = 7.1, H-9), 1.89 (m, 1H, H_eq_-22), 1.96 (m, 1H, H-4′), 1.97 (dd, 1H, ^3^*J*_18–19_ = 11.3, ^4^*J*_18–16eq_ = 1.8, H-18), 2.00 (m, 1H, H_ax_-16), 2.12 (ddd, 1H, ^2^*J* = 18.1, ^3^*J*_11ax-9_ = 10.6, ^3^*J*_11ax-12_ = 3.6, H_ax_-11), 2.22 (m, 1H, H_eq_-11), 2.24 (d, 1H, ^2^*J* = 14.9, H_A_-1), 2.60 (m, 1H, H*_trans_*-2′), 2.80 (d, 1H, ^2^*J* = 14.9, H_B_-1), 2.83 (m, 2H, H-7′), 2.90 (m, 1H, H_A_-6′), 2.98 (m, 1H, H_B_-6′), 3.33 (m, 1H, H*_cis_*-2′), 3.90 (m, 1H, H-3′), 5.45 (t, 1H, ^3^*J*_12–11ax_ = 3.6, ^3^*J*_12–11eq_ = 3.6, H-12), 6.29 (br.d, 1H, ^3^*J*_NH-3′_ = 6.7, NH), 7.05 (ddd, 1H, ^3^*J*_33–32_ = 7.6, ^3^*J*_33–34_ = 7.2, ^3^*J*_33–35_ = 1.1, H-33), 7.11 (ddd, 1H, ^3^*J*_34–35_ = 8.0, ^3^*J*_34–33_ = 7.2, ^3^*J*_34–32_ = 1.1, H-34), 7.30 (dd, 1H, ^3^*J*_35–34_ = 8.0, ^3^*J*_35–33_ = 1.1, H-35), 7.40 (dd, 1H, ^3^*J*_32–33_ = 7.6, ^3^*J*_32–34_ = 1.1, H-32), 7.97 (m, 1H, H-37). ^13^C NMR (CDCl_3_, δ ppm): 15.86 (C25), 17.07 (C26), 17.22 (C29), 19.29 (C6), 20.26 (C5′), 21.18 (C30), 23.18 (C27), 23.27 (C24), 23.64 (C11), 24.76 (C16), 25.50 (C4′), 25.66 (C8′), 28.06 (C15), 31.03 (C21), 31.05 (C23), 32.61 (C7), 34.05 (C4), 37.15 (C1), 37.63 (C22), 38.06 (C10), 39.20 (C20), 39.77 (C8), 39.93 (C19), 42.91 (C14), 46.11 (C3′), 46.30 (C9), 46.48 (C7′), 47.18 (C6′), 47.95 (C17), 53.27 (C5), 54.55 (C18), 56.33 (C2′), 106.71 (C2), 110.44 (C35), 117.87 (C32), 118.90 (C33), 120.97 (C34), 125.76 (C12), 128.23 (C31), 136.25 (C36), 139.98 (C13), 140.87 (C3), 178.06 (C28). *F1*-projection in {^1^H, ^15^N} HMBC (CDCl_3_, δ ppm): 21.70 (N1′), 117.05 (N37), 123.56 (NH). MW 635.98. C_43_H_61_N_3_O. Calculated, %: C 81.21; H 9.67; N 6.61.
*[3,2b]Indolo-11-dezoxo-olean-12-en-30-((±)-3-aminoquinuclidine) amide* (**12**). Yield 0.55 g (87%), brown powder, mp: 141.5 °C. ^1^H NMR (CDCl_3_, δ ppm, *J* Hz): 0.81 (s, 3H, H-28), 0.88 (m, 1H, H_eq_-16), 0.97 (s, 3H, H-25), 1.05 (m, 1H, H-26), 1.05 (m, 1H, H_eq_-15), 1.20 (s, 3H, H-27), 1.21 (s, 3H, H-29), 1.23 (s, 3H, H-24), 1.32 (s, 3H, H-23), 1.35 (m, 1H, H_ax_-22), 1.37 (m, 1H, H_eq_-22), 1.41 (m, 1H, H_ax_-21), 1.43 (dd, 1H, ^3^*J*_5–6ax_ = 10.8, ^3^*J*_5–6eq_ = 2.7, H-5), 1.46 (m, 1H, H_eq_-7), 1.56 (m, 1H, H_ax_-6), 1.63 (m, 1H, H_eq_-6), 1.63 (m, 1H, H_ax_-7), 1.66 (dd, 1H, ^2^*J* = 13.5, ^3^*J*_19ax-18_ = 13.5, H_ax_-19), 1.76 (m, 1H, H*_anti_*-5′), 1.82 (m, 1H, H_ax_-15), 1.86 (dd, 1H, ^3^*J*_9–11ax_ = 10.4, ^3^*J*_9–11eq_ = 7.1, H-9), 1.98 (m, 2H, H-8′), 1.99 (m, 1H, H_eq_-19), 2.00 (m, 1H, H_ax_-16), 2.03 (dd, 1H, ^3^*J*_18–19ax_ = 13.5, ^3^*J*_18–19eq_ = 4.1, H-18), 2.09 (m, 2H, H-11), 2.21 (d, 1H, ^2^*J* = 15.1, H_A_-1), 2.24 (m, 1H, H_eq_-21), 2.29 (m, 1H, H*_syn_*-5′), 2.39 (m, 1H, H-4′), 2.78 (d, 1H, ^2^*J* = 15.1, H_B_-1), 3.09 (m, 2H, H-7′), 3.19 (m, 1H, H_A_-6′), 3.39 (m, 1H, H*_trans_*-2′), 4.01 (m, 1H, H_B_-6′), 4.10 (m, 1H, H*_cis_*-2′), 4.42 (m, 1H, H-3′), 5.39 (t, 1H, ^3^*J*_12–11ax_ = 3.5, ^3^*J*_12–11eq_ = 3.5, H-12), 7.05 (ddd, 1H, ^3^*J*_33–32_ = 7.6, ^3^*J*_33–34_ = 7.2, ^3^*J*_33–35_ = 1.1, H-33), 7.10 (ddd, 1H, ^3^*J*_34–35_ = 8.0, ^3^*J*_34–33_ = 7.2, ^3^*J*_34–32_ = 1.1, H-34), 7.30 (dd, 1H, ^3^*J*_35–34_ = 8.0, ^3^*J*_35–33_ = 1.1, H-35), 7.41 (dd, 1H, ^3^*J*_32–33_ = 7.6, ^3^*J*_32–34_ = 1.1, H-32), 7.61 (br.d, 1H, ^3^*J*_NH-3′_ = 6.7, NH), 7.94 (m, 1H, H-37). ^13^C NMR (CDCl_3_, δ ppm): 15.77 (C25), 16.64 (C26), 17.75 (C5′), 19.41 (C6), 22.06 (C8′), 23.33 (C24), 23.68 (C11), 24.78 (C4′), 25.83 (C27), 26.21 (C15), 27.08 (C16), 28.41 (C28), 29.52 (C29), 31.03 (C23), 31.51 (C21), 32.05 (C17), 32.20 (C7), 34.05 (C4), 37.00 (C1), 38.06 (C10), 38.39 (C22), 39.93 (C8), 41.82 (C14), 43.29 (C19), 43.94 (C20), 44.58 (C3′), 46.27 (C7′), 46.30 (C6′), 46.37 (C9), 48.13 (C18), 50.57 (C2′), 53.27 (C5), 106.90 (C2), 110.39 (C35), 117.98 (C32), 118.88 (C33), 120.92 (C34), 122.90 (C12), 128.31 (C31), 136.22 (C36), 140.93 (C3), 144.17 (C13), 178.07 (C30). *F1*-projection in {^1^H, ^15^N} HMBC (CDCl_3_, δ ppm): 115.77 (NH), 116.76 (N37). MW 635.98. C_43_H_61_N_3_O. Calculated, %: C 81.21; H 9.67; N 6.61.5**.**

### 3.2. Biological Assays

#### 3.2.1. In Vitro AChE Inhibition Assay

Acetylcholinesterase from Electrophorus electricus (electric eel) (AChE, Sigma C2888), sodium phosphate buffer (PBS) 0.1 M pH 7.4, acetylthiocholine iodide (ACT, Sigma A5751), and 5,5′-dithiobis-(2-nitrobenzoic acid) (DTNB, Sigma D8130) were purchased from Sigma-Aldrich (St. Louis, MO, USA). In vitro anti-AChE activity was performed based on the modified Ellman’s method. For this purpose, synthesized compounds were dissolved in DMSO (20 mg/mL) and sodium phosphate buffer to final concentrations of 128, 32, 8, 2, and 0.5 µg/mL. In a 96-well plate, a mixture of 25 µL of prepared sample as described above, 25 µL PBS, 25 µL of 0.22 U/mL AChE, and 125 µL of 3 mM DTNB was pre-incubated for 15 min at 25 °C. Then, the substrate ACTI (25 µL of 15 mM ATCI) was added to the resulting mixture. Characterization of the hydrolysis of ATCI catalyzed by AChE was performed spectrometrically at 412 nm. The change in absorbance was measured at 412 nm after 15 min at 37 °C. The IC_50_ values were determined graphically from inhibition curves (log inhibitor concentration vs. percent of inhibition). All experiments were performed in triplicate. Donepezil was used as a positive control

#### 3.2.2. In Vitro AChE Inhibition Assay of Recombinant Human AChE

Acetylthiocholine iodide (ATC), recombinant human acetylcholinesterase (hAChE), and 5,5′-dithio-bis-(2-nitrobenzoic) acid (DTNB) were purchased from Sigma-Aldrich (Sigma-Aldrich). Dimethyl sulfoxide (DMSO) was purchased from PanReac AppliChem GmbH (Darmstadt, Germany). All other chemicals were of biochemical grade. All assays were performed at 25 °C using a UV-1800 Shimadzu (Kyoto, Japan) spectrophotometer at 412 nm. The inhibitory potency of compounds against hAChE was studied using Ellman’s method [27]. Stock solutions of compounds (0.01 M) were prepared using DMSO. The final concentration of DMSO in the cuvette was less than 0.05% vol. The inhibitory effect against AChE of 0.05% DMSO was considered weak [28]. To determine the IC_50_ (the concentration of the compound producing 50% enzyme activity inhibition), enzyme-catalyzed hydrolysis was carried out in 0.1 M phosphate buffer (pH 8.0) containing hAChE in the picomolar range, DTNB (0.1 mM final concentration), and 1 mM ATC as substrate. The tested compounds were pre-incubated with the enzyme for 5 min at 25 °C prior to the addition of the substrate. Then, absorbance change was recorded for 2 min. An enzyme mixture with 0.05% DMSO was used as a control. The sample without substrate was used as a blank. Experiments were performed in triplicate. The percentage of hAChE inhibition was determined by comparison of rates of reaction of test samples relative to the control sample. The percentage of inhibition as a function of compound concentration was plotted using OriginPro 8.5 software (OriginLab Corporation, Northampton, MA, USA). *IC*_50_ was calculated using the Hill Equation:

$$\frac{E}{E_{max}}=\frac{{\left[I\right]}^{n}}{IC_{50}^{n}+{\left[I\right]}^{n}}$$

where *E* is the enzyme activity and [*I*] is the compound concentration.

The inhibition constants for hAChE were determined in 0.1M sodium phosphate buffer (pH 8.0) with various ATC concentrations in the range 0.1–1 mM. The rate of hydrolysis of ATC was measured after the addition of DTNB (0.1 mM final concentration) and enzyme. The final enzyme concentration in the assays was in the picomolar range. Controls of the active enzyme with 0.05% DMSO and ATC spontaneous hydrolysis in the absence of an inhibitor were performed for each substrate concentration. The rate of substrate hydrolysis was recorded by monitoring the linear optical density change at 412 nm for 5 min. Experiments were performed in triplicate. Inhibition constants were determined from the Dixon plot and Cornish-Bowden transformation using OriginPro 8.5 software (OriginLab Corporation, Northampton, MA, USA) [29].

## 4. Conclusions

In summary, triterpenic 3-oxo- and 2,3-indole acids of lupane, oleanane, and ursane types were successfully conjugated with 3-aminoquinuclidine to yield corresponding amides. The biological evaluation showed that amides of 3-oxo-ursolic and 2,3-indolo-oleanolic acids exhibited inhibitory activity toward the AChE enzyme with IC_50_ 0.43 and 0.47 µM, respectively. Evaluation of the enzyme kinetics revealed that these compounds have a different mechanism of action. 3-Oxo-ursolic-3-aminoquinuclidine amide was identified as a mixed-type inhibitor, while its isomeric oleanolic amide with an indole fragment in the A-ring acted as an uncompetitive-type inhibitor.

## Figures and Tables

**Table 1:** IC_50_ values of compounds **5**–**12** against AChE enzyme.

Compound	IC_50_, µM	Compound	IC_50_, µM
**6**	17.00 ± 1.04	**10**	4.77 ± 0.21
**7**	1.67 ± 0.05	**11**	0.47 ± 0.03
**8**	0.43 ± 0.02	**12**	18.00 ± 1.39
**9**	28.86 ± 1.86	**13**	>128
		Donepezil	0.029 ± 0.003

**Table 2:** IC_50_ values of compounds **7** and **10** against hAChE enzyme.

Compound	IC_50_, µM	Compound	IC_50_, µM
**8**	5.33 ± 1.60	**11**	8.89 ± 0.40

**Table 3:** Inhibition constants for compounds **8** and **11**.

Compound	K_ci_, µM	K_ui_, µM
**8**	6.8 ± 0.4	22.8 ± 1.5
**11**	-	19.1 ± 5.1

## Data Availability

The data presented in this study are available on request from the corresponding author.

## Supplementary Materials

The following supporting information can be downloaded at: https://www.mdpi.com/article/10.3390/molecules30010095/s1, Figures S1–S16: ^1^H and ^13^C NMR spectra of compounds **5**–**12**.
