# Supplementary material for: Conjugation of Triterpenic Acids with 3-Aminoquinuclidine Moiety: An Approach to Acetylcholinesterase Mixed or Uncompetitive Type Inhibitors
Source: Molecules. 2024 Dec 29;30(1):95. doi: 10.3390/molecules30010095 (PMC11721041; doi:10.3390/molecules30010095)
Supplement: Supplementary file 1 [file molecules-30-00095-s001.zip › molecules-3376728-supplementary.pdf]

molecules

MDPI

Article

Conjugation of Triterpenic Acids with 3-Aminoquinuclidine Moiety: An Approach to Acetylcholinesterase Mixed or Uncompetitive Type Inhibitors

Anastasiya V. Petrova [1,*], Ha T. T. Nguyen [2], Irina V. Zueva [3], Konstantin A. Petrov [3], Alexander N. Lobov [1] and Oxana B. Kazakova [1]

[1] Ufa Institute of Chemistry, Ufa Federal Research Centre, Russian Academy of Science, 71, Prospect Octyabrya, Ufa 450054, Russia; lobovan@anrb.ru (A.N.L.); obf@anrb.ru (O.B.K.)
[2] Institute of Chemistry, Vietnam Academy of Science and Technology (VAST), 18, Hoang Quoc Viet Road, Cau Giay, Ha Noi 10000, Vietnam; thuha.vast@gmail.com
[3] Arbuzov Institute of Organic and Physical Chemistry, FRC Kazan Scientific Center, Russian Academy of Science, 8, Arbuzov Street, Kazan 420088, Russia; zueva.irina.vladimirovna@gmail.com (I.V.Z.); kpetrov2005@mail.ru (K.A.P.)
* Correspondence: ana.orgchem@gmail.com

Table of contents

^{1}H and ^{13}C NMR spectra of compounds **6-13**

^{1}H and ^{13}C NMR spectra of compound **6** 3

^{1}H and ^{13}C NMR spectra of compound **7** 4

^{1}H and ^{13}C NMR spectra of compound **8** 5

^{1}H and ^{13}C NMR spectra of compound **9** 6

^{1}H and ^{13}C NMR spectra of compound **10** 7

^{1}H and ^{13}C NMR spectra of compound **11** 8

^{1}H and ^{13}C NMR spectra of compound **12** 9

^{1}H and ^{13}C NMR spectra of compound **13** 10

S1. NMR spectra of compounds **6-13**

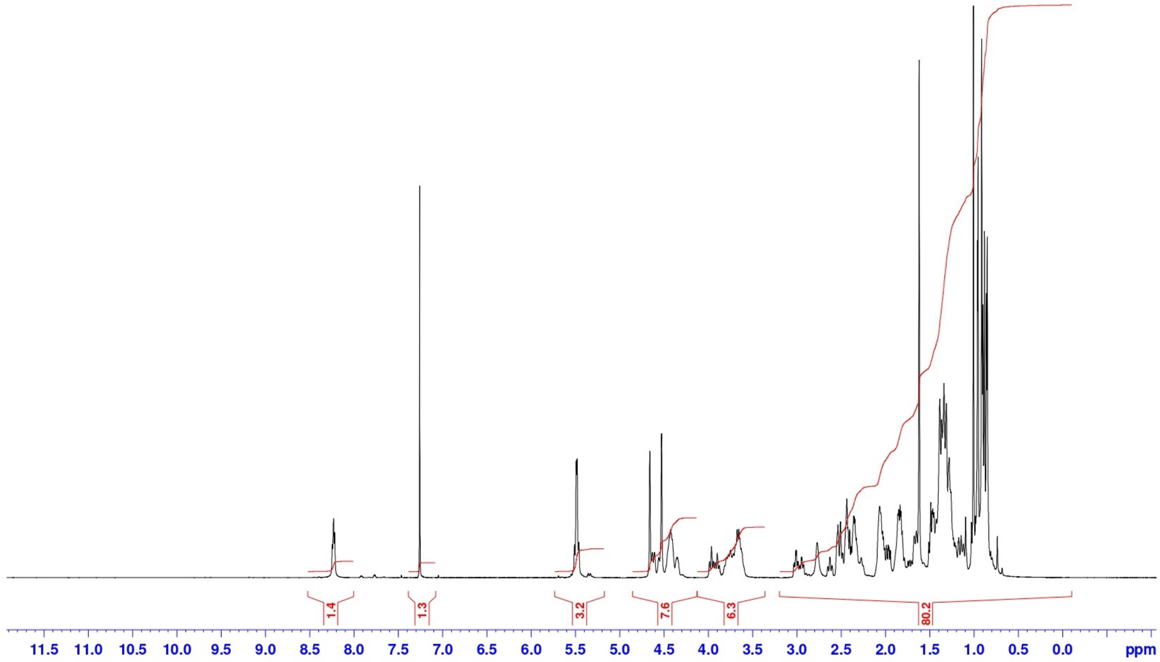

Figure S1. ^{1}H NMR spectrum of compound **6**

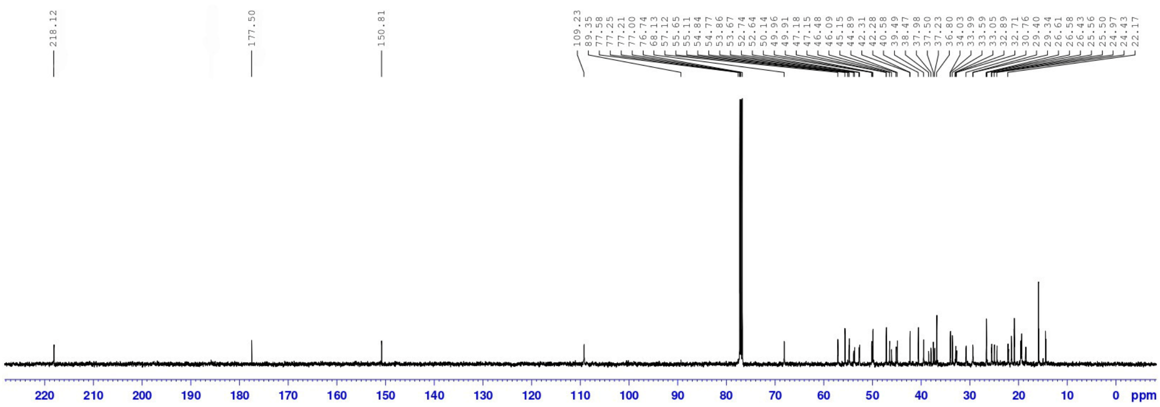

Figure S2. ^{13}C NMR spectrum of compound **6**

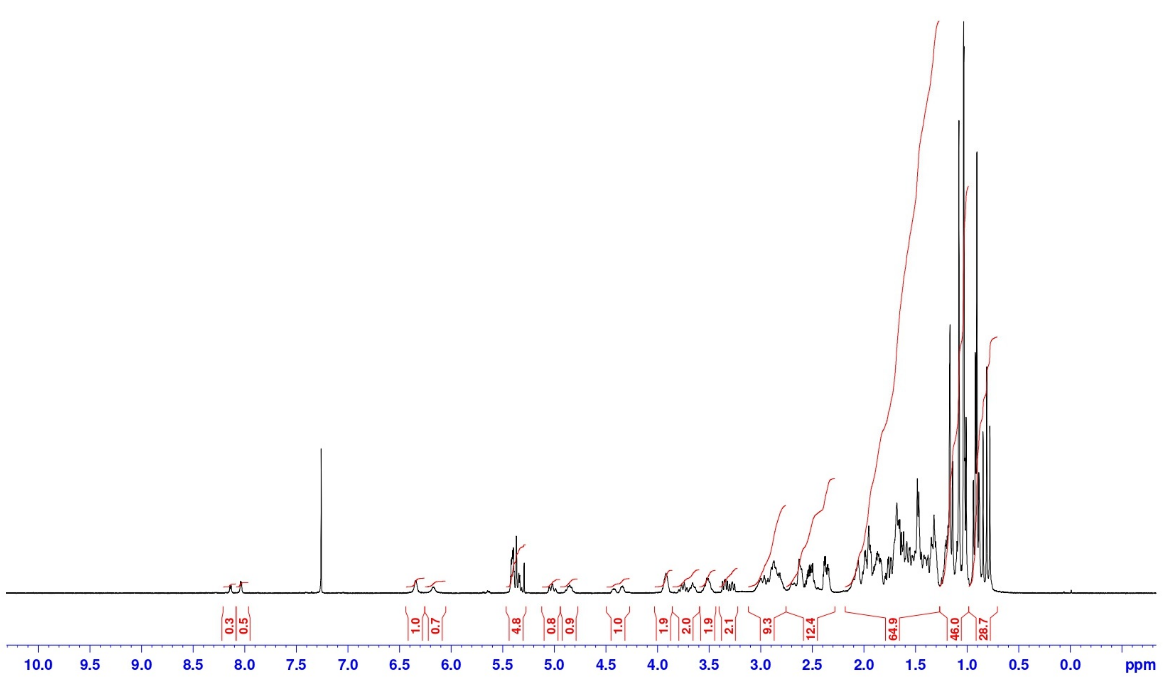

Figure S3. ^{1}H NMR spectrum of compound **7**

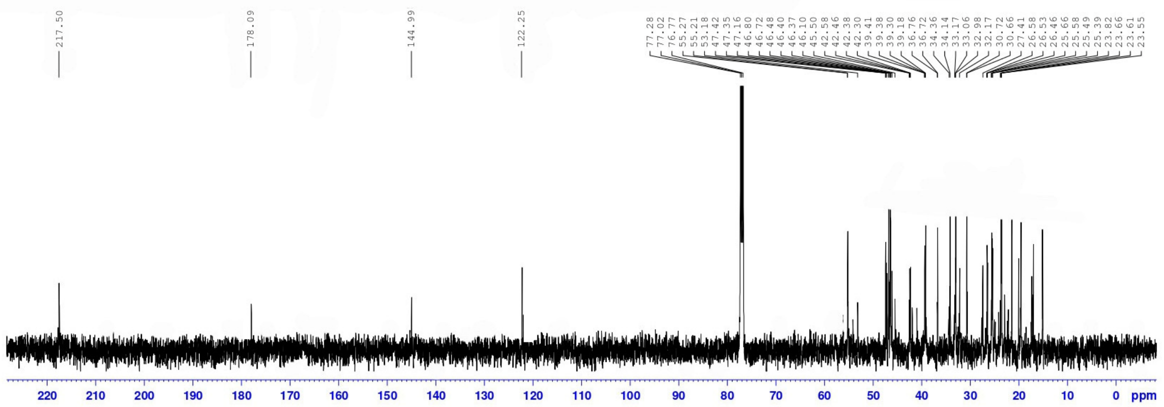

Figure S4. ^{13}C NMR spectrum of compound **7**

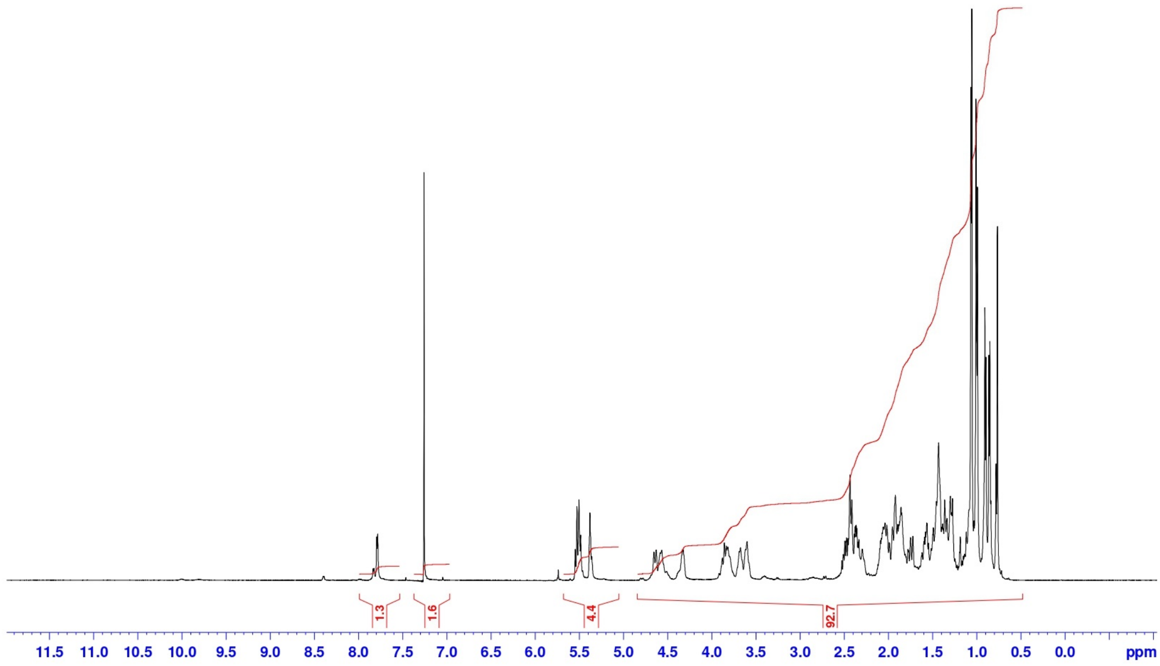

Figure S5. ^{1}H NMR spectrum of compound **8**

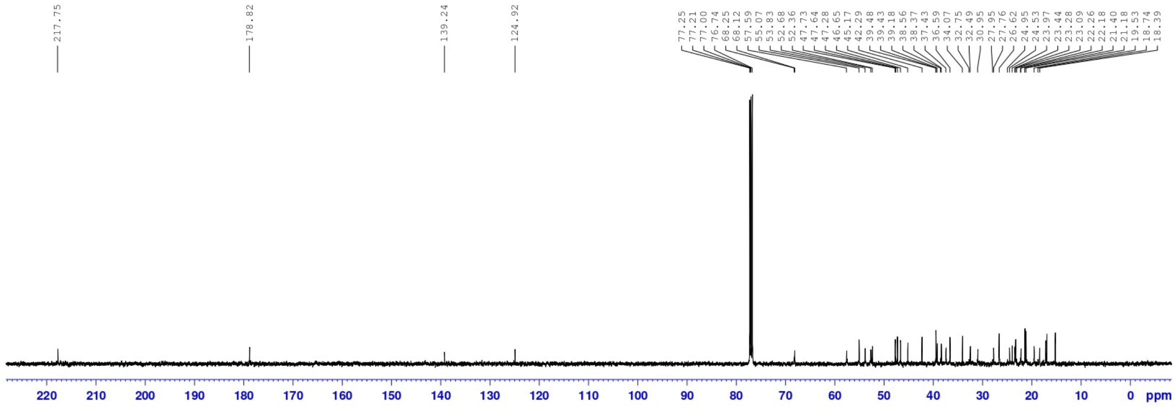

Figure S6. ^{13}C NMR spectrum of compound **8**

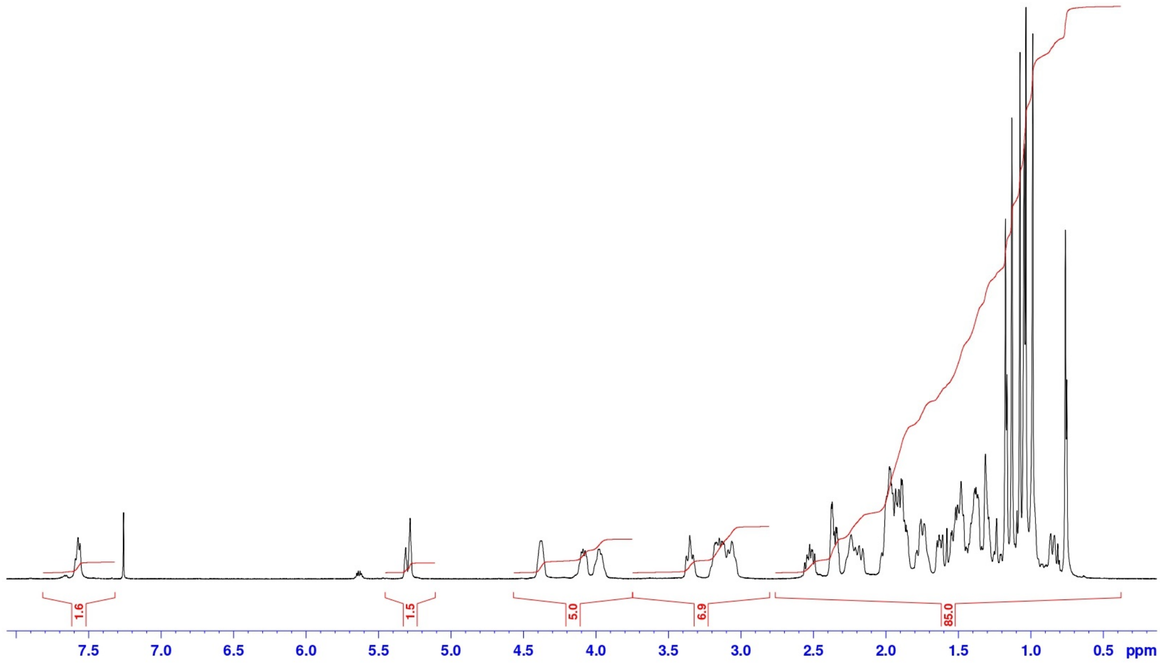

Figure S7. ^{1}H NMR spectrum of compound **9**

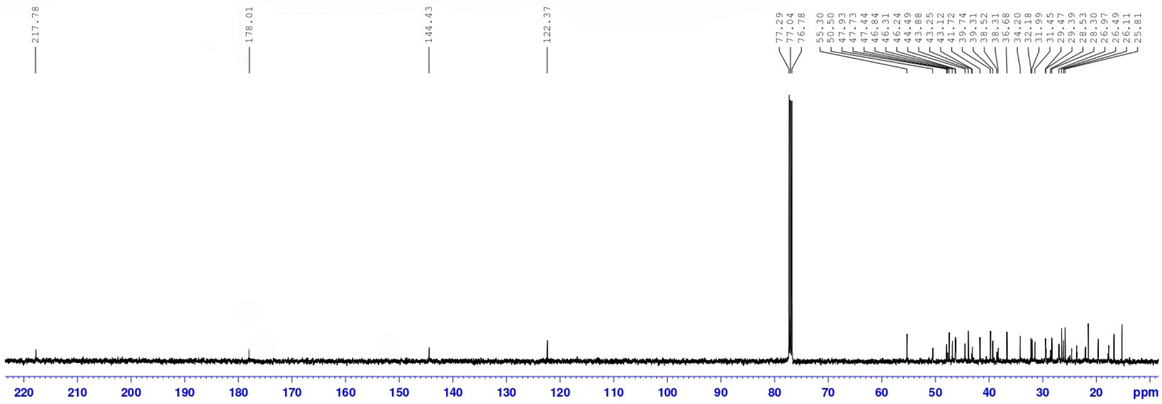

Figure S8. ^{13}C NMR spectrum of compound **9**

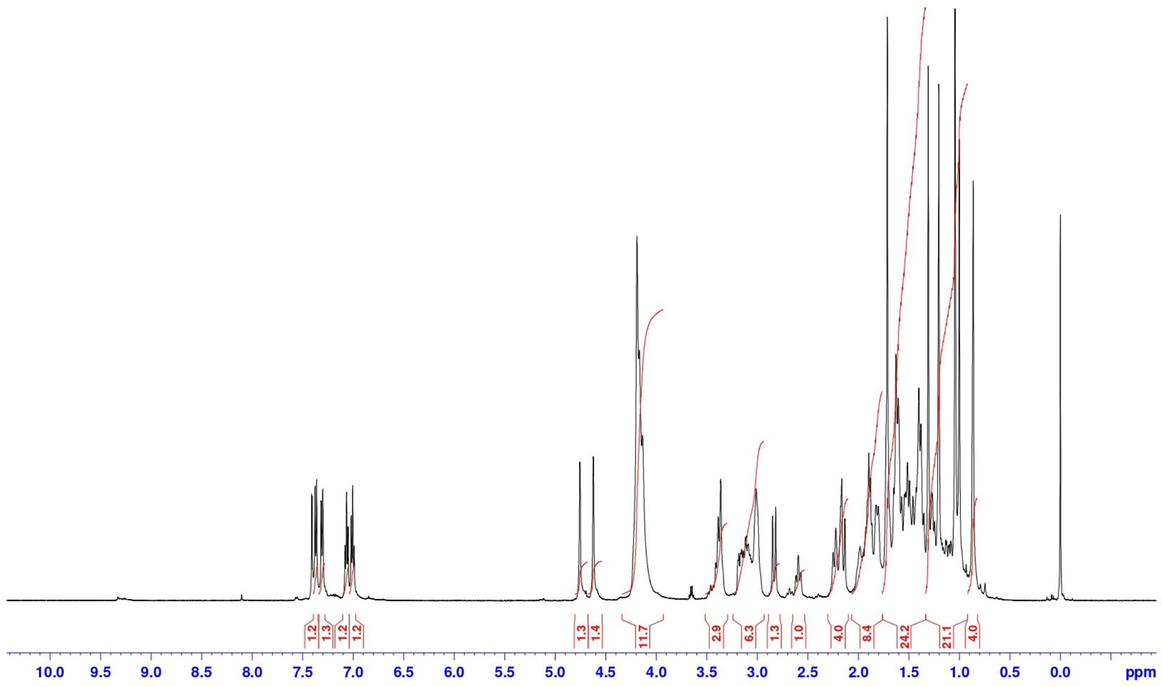

Figure S9. ^{1}H NMR spectrum of compound **10**

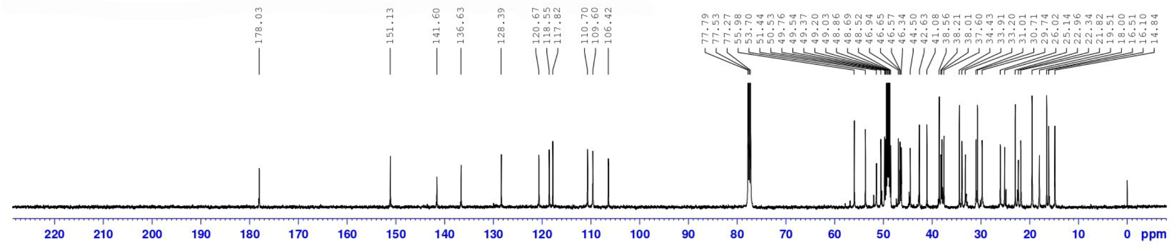

Figure S10 ^{13}C NMR spectrum of compound **10**

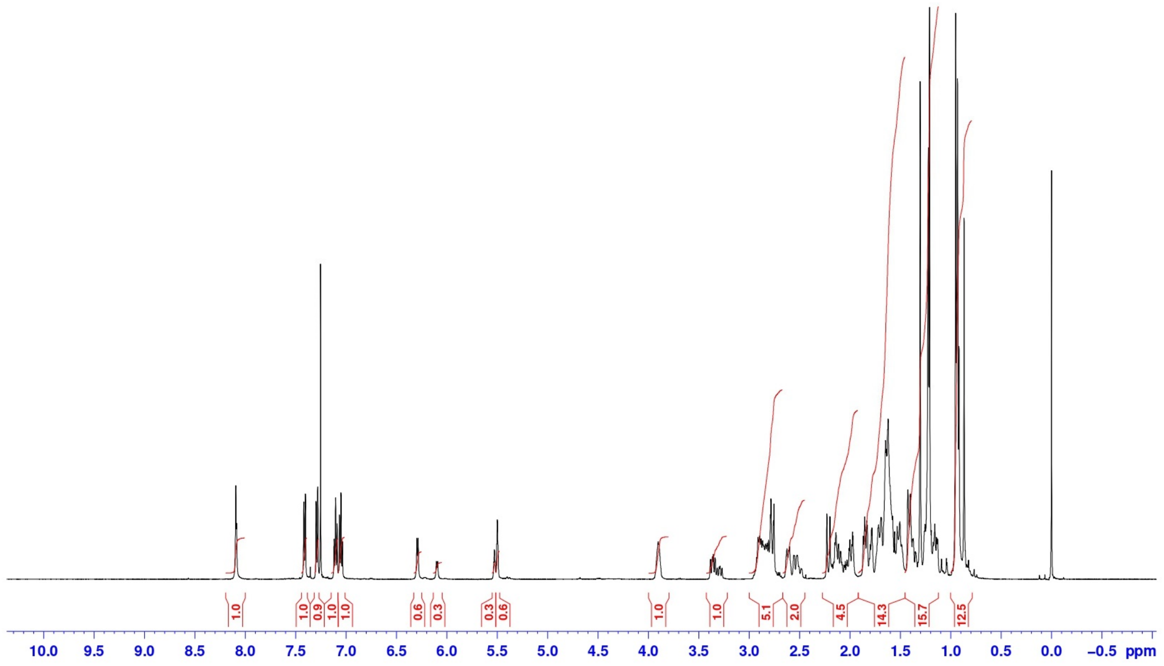

Figure S11. ^{1}H NMR spectrum of compound **11**

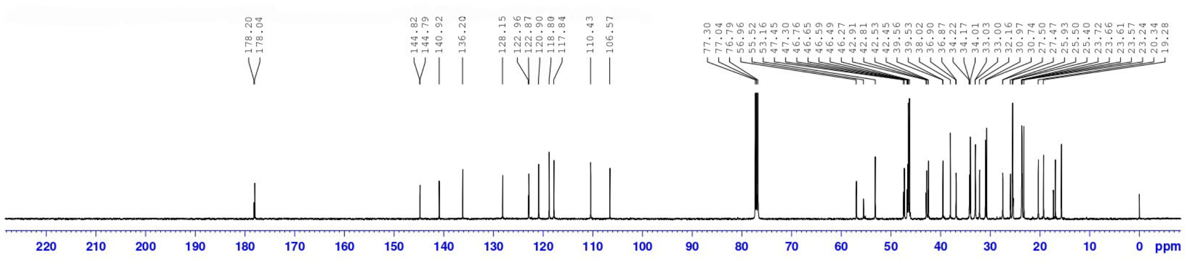

Figure S12. ^{13}C NMR spectrum of compound **11**

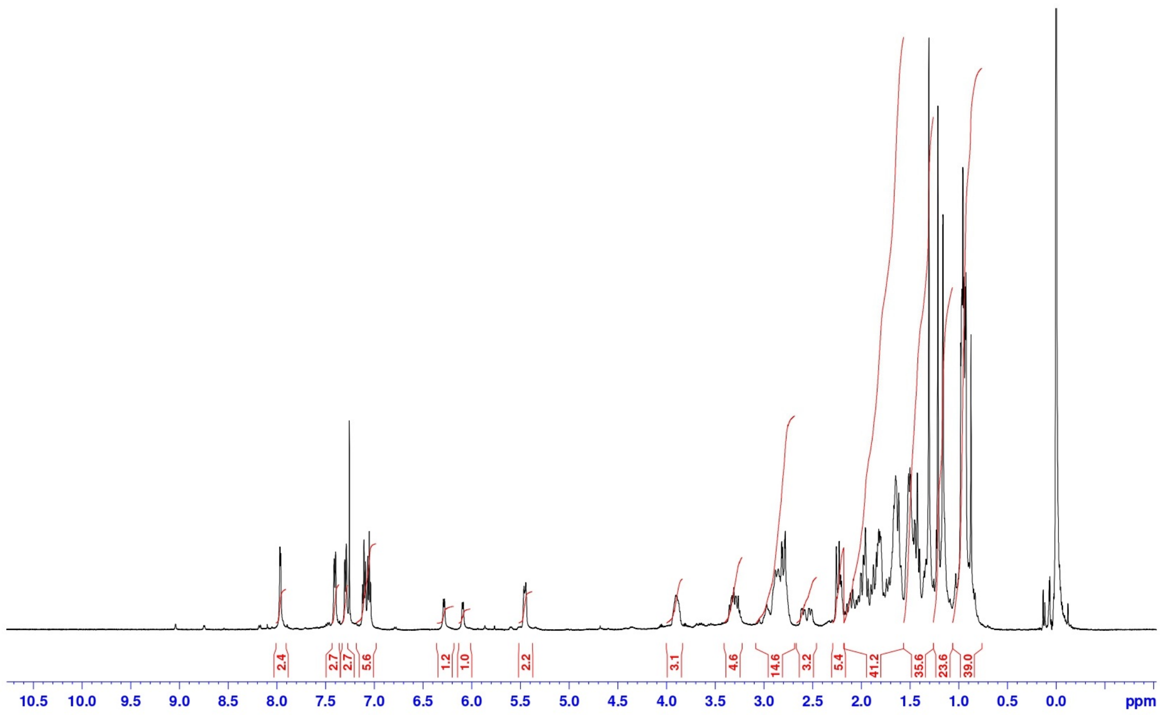

Figure S13. ^{1}H NMR spectrum of compound **12**

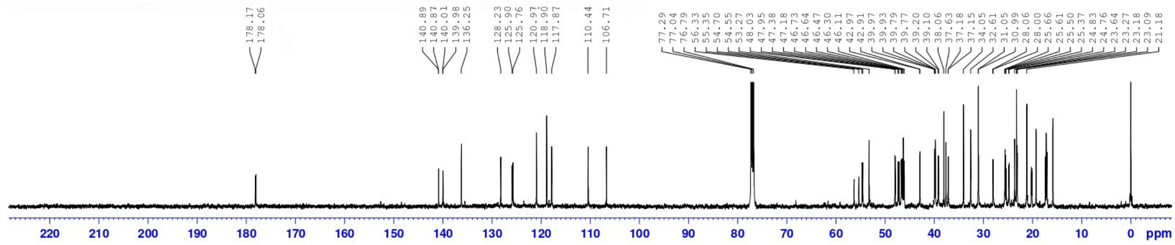

Figure S14. ^{13}C NMR spectrum of compound **12**

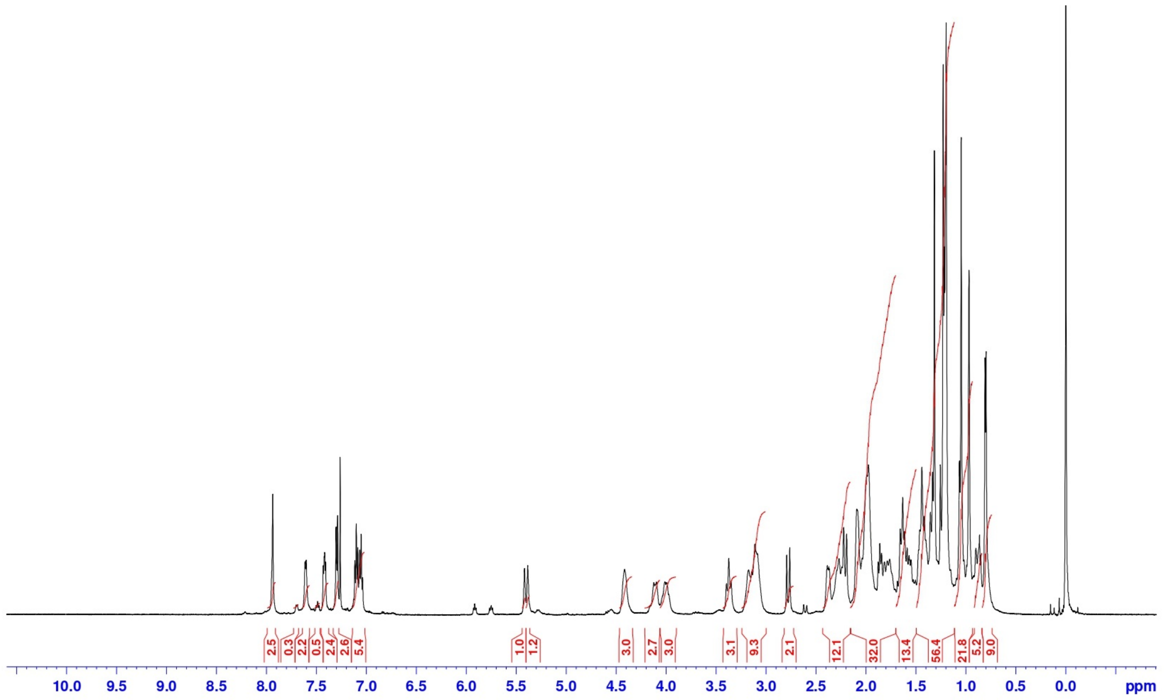

Figure S15. ^{1}H NMR spectrum of compound **13**

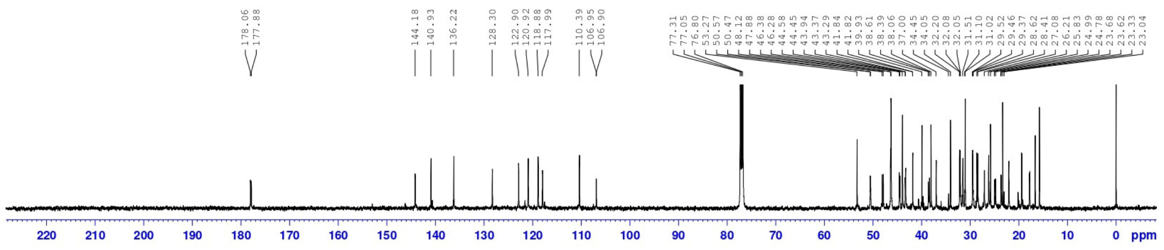

Figure S16. ^{13}C NMR spectrum of compound **13**
